# Hyperuricemia Predicts an Early Decline in Renal Function among Older People: A Community-Based Cohort Study

**DOI:** 10.1038/s41598-018-37529-z

**Published:** 2019-01-30

**Authors:** Wei-Cheng Tseng, Yung-Tai Chen, Yao-Ping Lin, Shuo-Ming Ou, Chih-Yu Yang, Chi-Hung Lin, Der-Cherng Tarng, The Taiwan Geriatric Kidney Disease (TGKD) Research Group, Der-Cherng Tarng, Wei-Cheng Tseng, Ming-Tsun Tsai, Shuo-Ming Ou, Chih-Yu Yang, Yao-Ping Lin, Yu-Hsin Chen, Yi-Fang Chuang, Liang-Kung Chen, Kwua-Yun Wang, Chia-Jen Shih, Yung-Tai Chen, Yi-Sheng Lin, Szu-Chun Hung, Ko-Lin Kuo, Tung-Po Hung, Fen-Hsiang Hu, Nien-Jung Chen, Yu-Chi Chen, Chi-Hung Lin, Tung-Hu Tsai, Shie-Liang Hsieh, Yau-Huei Wei, Chih-Cheng Hsu, Jia-Sin Liu, Yu-Kang Chang, Ming-Han Chiang

**Affiliations:** 1 Division of Nephrology, Department of Medicine, Taipei Veterans General Hospital, Taipei, Taiwan; 2 Division of Nephrology, Department of Medicine, Taipei City Hospital Heping-Fuyou Branch, Taipei, Taiwan; 3 Faculty of Medicine, National Yang-Ming University, Taipei, Taiwan; 4 Department and Institute of Physiology, National Yang-Ming University, Taipei, Taiwan; 5 Institute of Clinical Medicine, National Yang-Ming University, Taipei, Taiwan; 6 Institute of Microbiology and Immunology, National Yang-Ming University, Taipei, Taiwan; 7 Taipei Veterans General Hospital, Taipei, Taiwan; 8 Taipei Veterans General Hospital, Yuanshan Branch, Yilan, Taiwan; 9 Taipei City Hospital, Taipei, Taiwan; 10 Taipei Tzu Chi Hospital, Taipei, Taiwan; 11 Wei Gong Memorial Hospital, Miaoli, Taiwan; 12 National Yang-Ming University, Taipei, Taiwan; 13 National Health Research Institutes, Zhunan, Taiwan

## Abstract

Whether elevated serum uric acid levels (SUA) predict renal dysfunction remains controversial in the elderly. Therefore, we investigated the association between SUA and early renal function decline defined as an estimated glomerular filtration rate (eGFR) reduction ≥30% over 2 years. From 2001 to 2010, we conducted a longitudinal cohort study comprising 44,078 participants aged ≥65 years in the Taipei City Elderly Health Examination Database. Participants were classified by 1-mg/dL increment of SUA. We used multivariable logistic and Cox regression analyses to compare the risk of early renal function decline in different SUA groups. Compared to the reference SUA group of 5.0–5.9 mg/dL, hyperuricemic participants had increased risks of eGFR decline, starting at SUA ≥6.0 mg/dL (adjusted odds ratio [aOR] = 1.21, 95% confidence interval [CI] = 1.00–1.45). The risk progressively elevated as SUA increased, with the highest in the SUA ≥10.0 mg/dL group (aOR = 3.20, CI = 2.39–4.28). Multivariable Cox regression further confirmed that hyperuricemia was 1.12-fold (CI = 1.03–1.22, SUA ≥6.0 mg/dL) to 1.6-fold (CI = 1.37–1.86, SUA ≥10.0 mg/dL) more likely to develop early eGFR decline. Hyperuricemia-associated increased risks for early eGFR decline were consistent across subgroup and sensitivity analyses. Collectively, SUA ≥6.0 mg/dL independently predicted early renal dysfunction with eGFR decline ≥30% over 2 years in older people.

## Introduction

Older adults constitute the fastest-growing population of kidney diseases. Among individuals aged ≥65 years, chronic kidney disease (CKD) is a rather common disease, which affects 35% to 44% of the older people^1,2^. Mounting evidence has indicated that the elderly are at higher risks for CKD occurrence and progression, and early progressive renal function decline contributes to multiple serious adverse events, including cognitive impairment, end-stage kidney failure, cardiovascular disease and death^3,4^. Therefore, timely recognition of those older people at-risk for early renal function decline provides opportunities for prevention and treatment of adverse outcomes^5^. Traditional approach using a doubling of serum creatinine concentration (corresponding to a reduction of estimated glomerular filtration rate [eGFR] of 57% or greater) to document renal progression is a late event which precludes timely intervention^6^. A lesser reduction (≥30%) of eGFR over 2 years is a novel validated definition for early renal function decline and has been positively acknowledged as a surrogate endpoint of end-stage renal disease in clinical research^6–8^. Identification of predictors for early renal function decline defined as the eGFR decline ≥30% will help further promptly detect and treat older people at risks for advanced renal impairment.

Uric acid, the final product of purine degradation in humans, has been proposed to play pathophysiologic roles in renal diseases through inducing endothelial dysfunction and activating renin-angiotensin-aldosterone system^9^. Experimental studies indicate that hyperuricemia causes primary renal arteriolopathy in normal rats and accelerates progression of renal disease in the rat remnant kidney model^10–12^. Epidemiological studies also highlight that an elevated serum uric acid (SUA) level is associated with the development and progression of CKD in the middle-aged adults^13–17^. Nonetheless, whether hyperuricemia predicts deterioration of renal function in elderly population remains controversial^18–21^. Earlier studies suggest that elevated SUA levels are independently associated with a decline in eGFR of ≥3 mL/min/1.73 m^2^ per year in community-based healthy elderly cohorts^18,19^. Altemtam *et al*. also reported that baseline hyperuricemia independently predicted a faster renal progression of eGFR decline >2 ml/min/1.73 m^2^ per year in 270 elderly patients with stage 3 or 4 diabetic CKD^20^. By contrast, Nacak *et al*. recently found that SUA levels were not associated with the decline in renal function in stage 3 to 5 CKD patients^21^. These contradictory results may be ascribed to the different definitions of renal progression and reference SUA levels, relatively small sample size, varying characteristics of study population, and unequal statistical adjustment for confounders^18–21^. Several important confounding factors including smoking^21^, alcohol consumption^18–21^, history of cardiovascular diseases^18,19^, and fasting glucose levels^19–21^ are not fully adjusted previously. Furthermore, the reference SUA levels among these studies are unequal and most studies categorize participants by quantiles,^18,19^ which markedly limit the comparability and clinical applicability. Till now, there is no study identifying a threshold SUA level beyond which the risk of renal progression increases in the elderly population.

Therefore, the present study aimed to explore whether hyperuricemia independently predicted early renal function decline by utilizing a well-validated novel endpoint of eGFR decline ≥30% in a large community-based elderly cohort. We also intended to determine the range of optimal SUA levels with least risks of deteriorating renal function among older people.

## Methods

### Data Source

The present study was based on a large, well-characterized Taiwanese longitudinal community-based cohort of older people from the Taipei City Elderly Health Examination Database^22–24^, which contained health examination data for citizens 65 years or older since 2001, when the Taipei City Government launched an annual, free-of-charge, comprehensive health examination program to promote the health of senior citizens^25^. The accuracy of the Taipei City Elderly Health Examination Database was audited by Department of Health, Taipei City Government and the cohort has been validated previously^22,23^. Detailed information regarding height, weight, blood pressure, and prior medical history were recorded at the examination. Demographic data like age, gender, marital status, education levels, smoking history, alcohol consumption, and medical history including hypertension, diabetes mellitus, coronary artery disease and cerebrovascular disease, were collected through a self-administered questionnaire. Overnight fasting blood was collected for the measurement of complete blood count, glucose, triglyceride, total cholesterol, high-density-lipoprotein cholesterol, albumin, blood urea nitrogen, creatinine, and SUA. The SUA level was assayed by the colorimetric uricase–peroxidase system^26^. A semiquantitative (negative, trace, 1+, 2+, 3+, 4+) urine dipstick test for albuminuria was also performed. The Chronic Kidney Disease Epidemiology Collaboration (CKD-EPI) equation was used to calculate eGFR, which more accurately estimates GFR and predicts the risks of end-stage renal disease and mortality than the Cockcroft-Gault and Modification of Diet in Renal Disease Study equations^27,28^. All participants provided informed consent authorizing the Taipei City Government to process the health examination data for research purposes. Detailed information on the health examination data is stored centrally in the Taipei City Elderly Health Examination Database and is de-identified before release to protect the privacy. The present study was approved by the institutional review board of the Taipei City Hospital (TCHIRB-1010323-E and TCHIRB-1030601-W).

### Study Design and Participants

The study cohort comprised older adults participating in the Taipei City Elderly Health Examination Program with follow-up laboratory data from 2001 to 2010. Participants with repeated measurement of renal function were initially screened (n = 56,062). We excluded participants with missing measurements of SUA (n = 51), with the history of end-stage renal disease (n = 1,629), or with extremely low SUA levels (<2 mg/dL, n = 186) that could possibly be attributed to hereditary renal hypouricemia^29^. Those with follow-up periods less than 2 years (n = 2,854) or without regular follow-up (n = 7,264) were also excluded. Finally, 44,078 older people were enrolled in the present study. To identify the optimal range of SUA levels with the least risks for early renal function decline, eligible participants were further classified by 1-mg/dL increment of SUA and participants with the SUA of 5.0–5.9 mg/dL were set as the reference group, because the relation between SUA levels and new-onset kidney disease has been observed in those with SUA levels more than 6.0 mg/dL in the general population^13^.

### Study Outcome and Follow-up

The study outcome was early renal function decline defined as the occurrence of ≥30% reduction in eGFR. Percent change in eGFR was calculated as (last eGFR at the follow-up period – baseline eGFR)/(baseline eGFR) × 100%^6,8^. All participants were followed until death or December 31, 2010, whichever occurred first.

### Statistical Analysis

Categorical and continuous variables were expressed as percentages and median (interquartile range), respectively. Between-group comparisons were made by the Kruskal-Wallis test or Pearson χ^2^ test where appropriate. Multivariable logistic regression was used to assess the association between baseline SUA levels and the study outcome because the original definition of early renal progression proposed by Coresh *et al*. is calculated at the end of a 2-year follow-up^6^. Covariates including age, sex, body mass index, smoking, alcohol consumption, systolic and diastolic blood pressure, hypertension, diabetes mellitus, dyslipidemia, coronary artery disease, cerebrovascular disease, baseline kidney function, plasma total cholesterol, triglyceride, high-density lipoprotein-cholesterol, hemoglobin, glucose, white blood cell count, and albumin were adjusted. Missing data of a certain baseline biochemical variable was replaced with the mean value of the same variable.

As SUA levels are inversely correlated with baseline kidney function, we also examined the predictive effect of SUA levels on eGFR decline in pre-specified stratified analyses according to baseline kidney function (eGFR >90, 60–90, 45–60 and <45 mL/min/1.73 m^2^). Consistency of the predictive effect of SUA levels on eGFR decline was further assessed in the subgroups based on age (<80 years and ≥80 years), gender, hypertension, diabetes, and coronary artery disease. Potential interactions between SUA levels and subgroups were explored by the likelihood ratio test. To further test the robustness of our findings, sensitivity analyses by assessing the association between SUA levels and the eGFR decline ≥30% over 1-year, 3-year, and 5-year follow-up periods, changing the threshold of eGFR decline to 40%, regrouping the participants into SUA deciles, and excluding participants who had albuminuria or baseline eGFR <60 mL/min/1.73 m^2^, participants who took urate-lowering agents, or participants who had missing value of other baseline biochemical covariates, were also performed. A cubic spline model was also constructed to describe the continuous association between SUA levels with early renal function decline, with three knots defined at SUA of 4.0 mg/dL, 6.0 mg/dL and 8.0 mg/dL. To further validate the association between hyperuricemia and early renal function decline, time-to-event analyses including Cox proportional hazards regression and competing-risk analysis by the Fine-Gray model, treating death as a competing risk event were also tested. In these time-to-event analyses, the study outcome was defined as an eGFR decline ≥30%. A two-tailed *p* value < 0.05 was considered significant. All analyses were conducted using the statistical software Stata (version 13.0; Stata Corp., College Station, TX).

## Results

### Baseline Characteristics

A total of 44,078 eligible participants (23,202 men and 20,876 women) were identified in the database from 2001 to 2010 (Supplemental Figure S1, Table 1). The median age of the study cohort was 71 (interquartile range, 9) years with 52.6% being male. Compared to the reference group (SUA level: 5.0–5.9 mg/dL), increasing SUA levels were associated with the higher age, body mass index, systolic and diastolic blood pressure, fasting plasma glucose and triglyceride levels, leukocyte count, and the percentage of male gender, smoking, alcohol use, hypertension, coronary artery disease, and cerebrovascular disease, but with lower baseline eGFR and high-density lipoprotein cholesterol levels (Table 1).

**Table 1:** Demographic and clinical characteristics of study population by serum uric acid levels*.

Characteristics	All	Serum Uric Acid (mg/dL)									*P* value
		2.0–2.9	3.0–3.9	4.0–4.9	5.0–5.9	6.0–6.9	7.0–7.9	8.0–8.9	8.0–9.9	≥10	
Number of participants	44,078	522	2,656	7,708	11,076	10,047	6,435	3,387	1,344	903	
Demographics											
Male	23,202 (52.6)	165 (31.6)	713 (26.8)	2,577 (33.4)	5,179 (46.8)	6,070 (60.4)	4,431 (68.9)	2,451 (72.4)	966 (71.9)	650 (72.0)	<0.001
Age, years	71 (9)	71 (9)	69 (9)	69 (9)	70 (10)	71 (9)	72 (9)	72 (9)	73 (8)	74 (8)	<0.001
Smoking	3,718 (8.4)	22 (4.2)	124 (4.7)	440 (5.7)	796 (7.2)	940 (9.4)	735 (11.4)	382 (11.3)	158 (11.8)	121 (13.4)	<0.001
Alcohol use	6,200 (14.1)	40 (7.7)	227 (8.5)	773 (10.0)	1,501 (13.6)	1,585 (15.8)	1,156 (18.0)	592 (17.5)	195 (14.5)	131 (14.5)	<0.001
BMI, kg/m^2^	24.0 (4.2)	22.6 (4.4)	22.8 (4.3)	23.2 (4.1)	23.7 (4.1)	24.3 (4.0)	24.7 (4.1)	25.0 (4.1)	25.1 (4.4)	25.2 (4.5)	<0.001
Comorbidity											
Hypertension	23,352 (53.0)	238 (45.6)	1,220 (45.9)	3,551 (46.1)	5,614 (50.7)	5,432 (54.1)	3,727 (57.9)	2,089 (61.7)	859 (63.9)	622 (68.9)	<0.001
Diabetes	4,560 (10.3)	63 (12.1)	311 (11.7)	801 (10.4)	1,133 (10.2)	1,019 (10.1)	648 (10.1)	329 (9.7)	162 (12.1)	94 (10.4)	0.086
Dyslipidemia	22,584 (51.2)	269 (51.5)	1,382 (52.0)	4,136 (53.7)	5,761 (52.0)	5,050 (50.3)	3,182 (49.4)	1,673 (49.4)	682 (50.7)	449 (49.7)	<0.001
CAD	4,775 (10.8)	52 (10.0)	256 (9.6)	751 (9.7)	1,051 (9.5)	1,105 (11.0)	806 (12.5)	449 (13.3)	172 (12.8)	133 (14.7)	<0.001
CVD	340 (0.8)	5 (1.0)	24 (0.9)	48 (0.6)	83 (0.7)	77 (0.8)	51 (0.8)	27 (0.8)	18 (1.3)	7 (0.8)	0.353
Blood pressure, mmHg											
Systolic	133 (27)	130 (24)	130 (27)	131 (25)	133 (26)	134 (27)	135 (27)	136 (26)	137 (27)	138 (26)	<0.001
Diastolic	76 (15)	76 (13)	75 (14)	75 (15)	76 (14)	77 (14)	77 (15)	79 (16)	78 (16)	79 (16)	<0.001
eGFR, mL/min/1.73 m^2^											<0.001
≥90	4,920 (11.2)	101 (19.3)	645 (24.3)	1,576 (20.4)	1,456 (13.1)	764 (7.6)	262 (4.1)	77 (2.3)	28 (2.1)	11 (1.2)	
60–89	25,120 (57.0)	323 (61.9)	1,533 (57.7)	4,687 (60.8)	6,939 (62.6)	6,034 (60.1)	3,438 (53.4)	1,448 (42.8)	479 (35.6)	239 (26.5)	
45–59	10,466 (23.7)	80 (15.3)	401 (15.1)	1,196 (15.5)	2,167 (19.6)	2,537 (25.3)	2,016 (31.3)	1,272 (37.6)	466 (34.7)	331 (36.7)	
30–44	2,892 (6.6)	12 (2.3)	65 (2.4)	206 (2.7)	443 (4.0)	590 (5.9)	565 (8.8)	476 (14.1)	296 (22.0)	239 (26.5)	
15–29	526 (1.2)	5 (1.0)	5 (0.2)	24 (0.3)	46 (0.4)	93 (0.9)	124 (1.9)	92 (2.7)	65 (4.8)	72 (8.0)	
<15	154 (0.3)	1 (0.2)	7 (0.3)	19 (0.2)	25 (0.2)	29 (0.3)	30 (0.5)	22 (0.6)	10 (0.7)	11 (1.2)	
Total cholesterol, mg/dL	198 (47)	198 (45)	199 (47)	201 (47)	199 (48)	197 (46)	195 (47)	195 (49)	196 (49)	195 (52)	<0.001
Triglyceride, mg/dL	106 (72)	84.5 (56)	91 (56)	96 (60)	103 67)	112 (76)	119 (77)	125 (83)	133 (89)	139 102)	<0.001
HDL-cholesterol, mg/dL	49 (18)	55 (26)	55.2 (25)	54 (22)	50 (18)	47.3 (15)	46.1 (13)	46 (12)	45 (10)	44 (9)	<0.001
WBC count, /mm^3^	5780 (1890)	5460 (1900)	5400 (1855)	5500 (1800)	5700 (1720)	5800 (1820)	5990 (1900)	6110 (1920)	6105 (2115)	6450 (2160)	<0.001
Albumin, g/dL	4.3 (0.4)	4.3 (0.4)	4.3 (0.4)	4.3 (0.4)	4.3 (0.4)	4.3 (0.4)	4.3 (0.4)	4.4 (0.4)	4.4 (0.4)	4.3 (0.4)	<0.001
Hemoglobin, g/dL	13.6 (1.7)	13.1 (1.6)	1.31 (1.4)	13.3 (1.6)	13.5 (1.6)	13.7 (1.7)	13.9 (1.8)	13.9 (1.9)	13.8 (2.1)	13.6 (2.2)	<0.001
Fasting glucose, mg/dL	99 (18)	97 (16)	97 (17)	98 (17)	99 (17)	99 (18)	101 (19)	101 (19)	102 (21)	102 (22)	<0.001

*Values for categorical variables are given as number (percentage); values for continuous variables are given as median (interquartile range).

Abbreviations: BMI, body mass index; CAD, coronary artery disease; CVD, cerebrovascular disease; eGFR, estimated glomerular filtration rate; HDL, high-density lipoprotein; SD, standard deviation; WBC, white blood cell

SI conversion factors: To convert uric acid value to μmol/L, multiply by 59.485; blood pressure value to pascal, multiply by 133.3; cholesterol (total or HDL-cholesterol) value to mmol/L, multiply by 0.0259; triglyceride value to mmol/L, multiply by 0.0113; WBC count to ×10^9^/L, multiply by 0.001; albumin value to g/L, multiply by 10; hemoglobin value to g/L, multiply by 10; glucose value to mmol/L, multiply by 0.055.

### Association between SUA levels and the risk of ≥30% eGFR decline in the elderly

There were 1190 participants who experienced early renal function decline with a ≥30% reduction in eGFR over 2 years. In multivariable analyses, hyperuricemic older people had increased risks for early renal function decline, starting at SUA ≥6.0 mg/dL (adjusted odds ratio [aOR] = 1.21, 95% confidence interval [CI] = 1.00–1.45), after adjustment for 20 demographic and clinical variables (Table 2). Notably, the risks for eGFR decline paralleled the increasing SUA levels with the highest risk in SUA group ≥ 10 mg/dL (aOR = 3.35, CI = 2.45–4.59). Remarkably, low SUA levels <3.0 mg/dL were also associated with a higher risk of eGFR decline (aOR = 1.69, CI = 1.08–2.63). After adjusting for proteinuria (dipstick trace or ≥1 + ), hyperuricemia still significantly predicted higher risks for early renal progression (Supplemental Table S1). In attempt to further decipher whether hyperuricemia induced renal dysfunction, the association between SUA levels and eGFR decline ≥30% over 2 years was tested in the participants free from CKD. Hyperuricemia remained significantly associated with early renal function decline in the group excluding participants with positive proteinuria or baseline eGFR <60 mL/min/1.73 m^2^ (Supplemental Table S1).

**Table 2:** Incidence and risks of eGFR decline ≥30% over a 2-year follow-up period in older people.

Serum uric acid (mg/dL)	Incidence		Logistic Regression Analysis			
	No. of Events	No. of Participants	Crude Odds Ratio (95% CI)	*P*	Adjusted Odds Ratio (95% CI)^a^	*P*
2.0–2.9	23 (4.4%)	522	2.07 (1.34–3.21)	0.001	1.69 (1.08–2.63)	0.021
3.0–3.9	64 (2.4%)	2,656	1.11 (0.84–1.47)	0.463	0.91 (0.68–1.20)	0.491
4.0–4.9	205 (2.7%)	7,708	1.23 (1.02–1.48)	0.032	1.10 (0.91–1.33)	0.319
5.0–5.9	241 (2.2%)	11,076	Reference		Reference	
6.0–6.9	233 (2.3%)	10,047	1.07 (0.89–1.28)	0.483	1.21 (1.00–1.45)	0.048
7.0–7.9	220 (3.4%)	6,435	1.59 (1.32–1.92)	<0.001	1.91 (1.58–2.32)	<0.001
8.0–8.9	103 (3.0%)	3,387	1.41 (1.12–1.78)	0.004	1.77 (1.38–2.26)	<0.001
9.0–9.9	41 (3.1%)	1,344	1.41 (1.01–1.98)	0.043	1.61 (1.13–2.29)	0.008
≥10	60 (6.6%)	903	3.20 (2.39–4.28)	<0.001	3.35 (2.45–4.59)	<0.001

^a^Adjusted for age, sex, body mass index, smoking, alcohol drinking, comorbidities and all biochemical data in Table 1.

Abbreviation: CI, confidence interval; eGFR, estimated glomerular filtration rate.

SI conversion factors: To convert uric acid value to μmol/L, multiply by 59.485.

### Hyperuricemia and the risk of ≥30% eGFR decline according to baseline renal function

**Figure 1:**
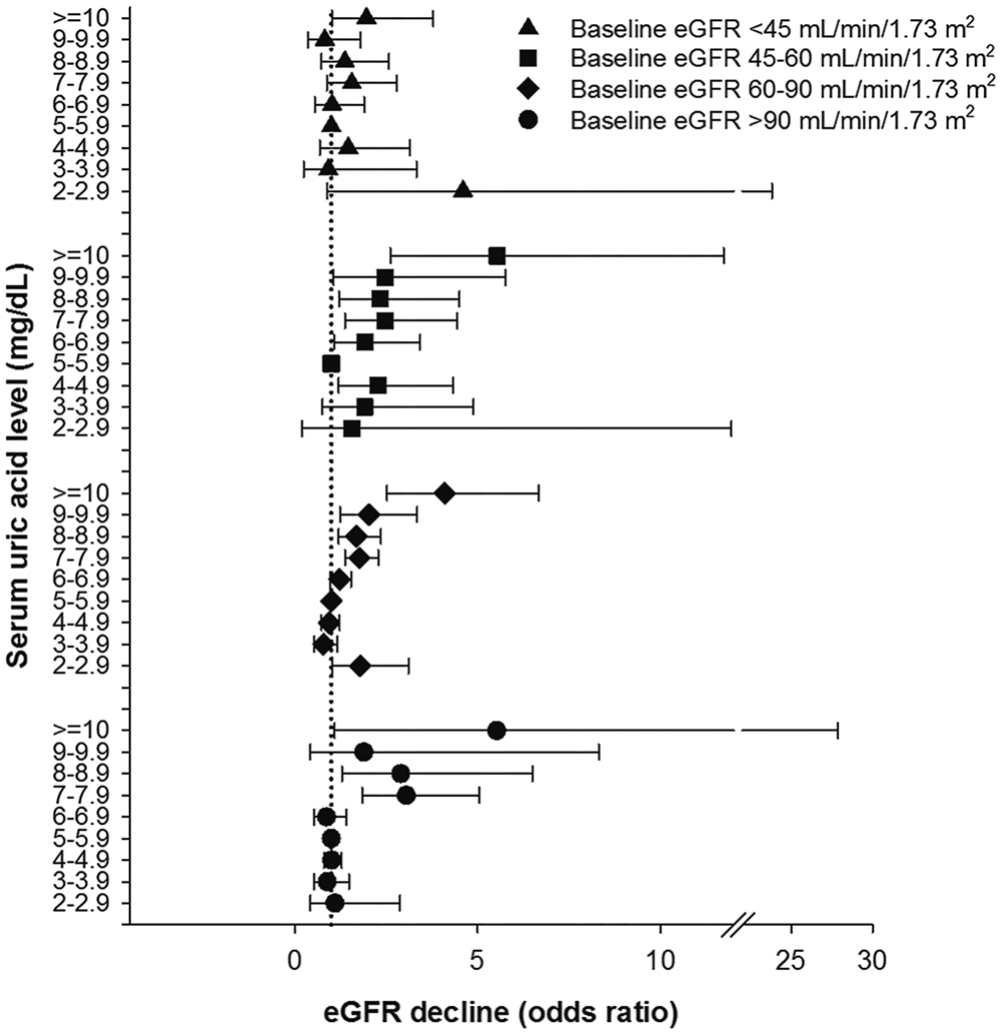
Association between serum uric acid levels and risks of early renal function decline stratified by baseline estimated glomerular filtration rate (eGFR). Odds ratios were calculated by multivariable logistic regression after adjusting for 20 demographic and clinical variables. Serum uric acid levels of 5.0–5.9 mg/dL served as the reference group. Bars denote 95% confidence intervals.

As SUA levels are inversely correlated with baseline kidney function, we also examined the effect-modifying role of baseline eGFR on hyperuricemia-associated early renal function decline. In the strata of eGFR >90 mL/min/1.73 m^2^ (aOR = 3.05, CI = 1.84–5.04), 60–90 mL/min/1.73 m^2^ (aOR = 1.77, CI = 1.37–2.28), and 45–60 mL/min/1.73 m^2^ (aOR = 2.47, C = 1.37–4.44), hyperuricemia was significantly associated with increased risks toward early eGFR decline once SUA levels exceeded 7.0 mg/dL. In the strata of baseline eGFR <45 mL/min/1.73 m^2^, risks of hyperuricemia-associated early renal function decline were significantly higher only when SUA levels surpassed 10.0 mg/dL (aOR = 1.96, CI = 1.02–3.79) (Fig. 1).

**Figure 2:**
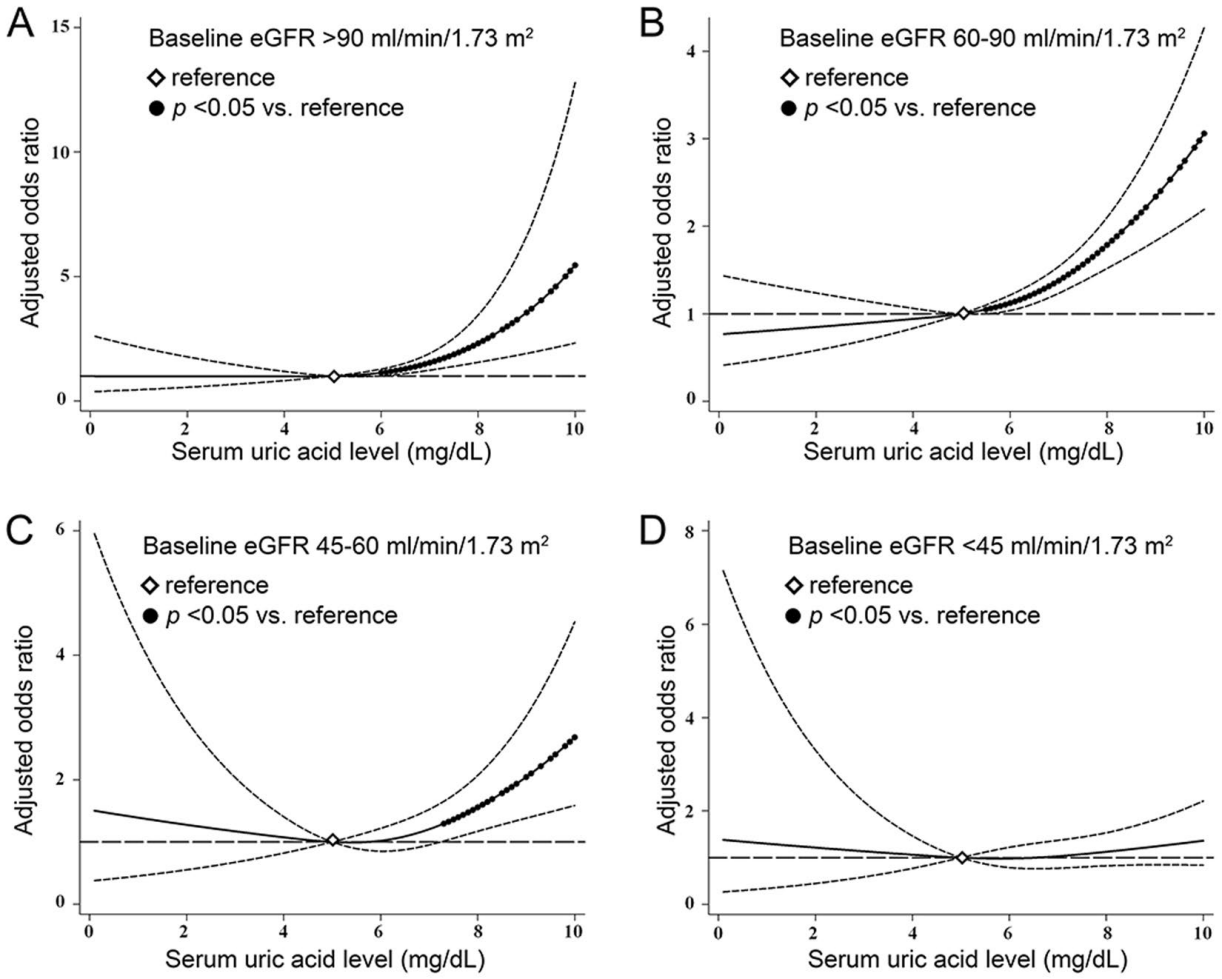
Cubic spline models for the association of serum uric acid levels with the risks of early renal function decline among strata of baseline eGFR (**A**) >90 ml/min/1.73 m^2^, (**B**) 60–90 ml/min/1.73 m^2^, (**C**) 45–60 ml/min/1.73 m^2^, and (**D**) <45 ml/min/1.73 m^2^. Models were adjusted for 20 demographic and clinical variables. Filled circles denote statistical significance (*p* < 0.05) compared to the reference (diamond) serum uric acid level of 5.0 mg/dL. Solid line (—) denotes adjusted odds ratio and dash line (—) denotes 95% confidence intervals.

In order to offer more insight on the association between SUA levels and early renal function decline, SUA levels were treated as continuous variables and analyzed by cubic spline models with the reference at SUA of 5.0 mg/dL. Higher SUA levels were consistently associated with elevated risks of early renal function decline in the baseline eGFR >90, 60–90 and 45–60 ml/min/1.73 m^2^ strata. The risks of eGFR decline started to significantly increase once SUA levels went beyond 6.0 mg/dL in the baseline eGFR >90 ml/min/1.73 m^2^ strata, beyond 5.5 mg/dL in the baseline eGFR 60–90 ml/min/1.73 m^2^ strata, and beyond 7.3 mg/dL in the baseline eGFR 45–60 ml/min/1.73 m^2^ strata (Fig. 2).

### Subgroup and sensitivity analysis of hyperuricemia-associated ≥30% eGFR Decline

**Figure 3:**
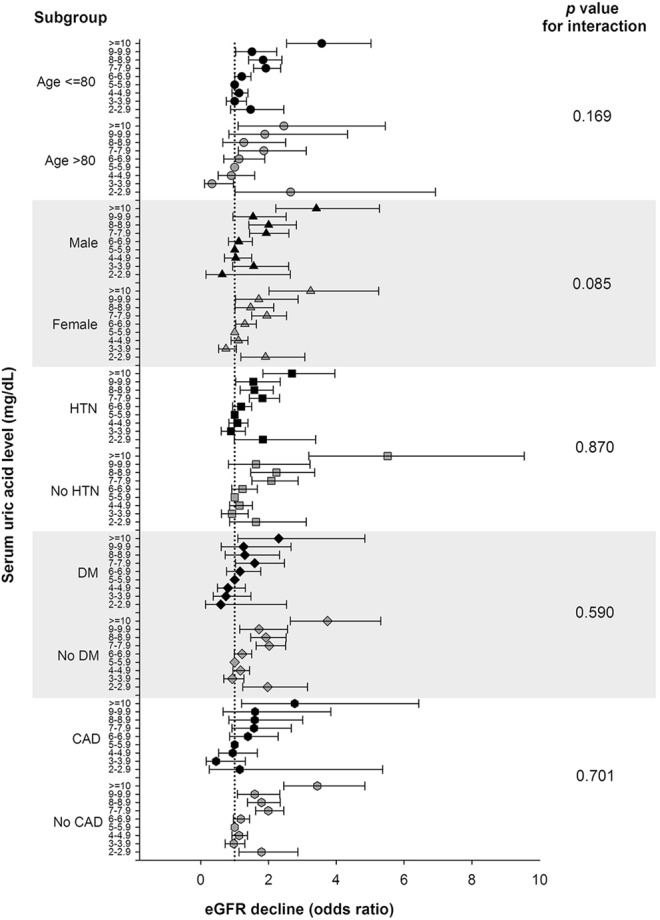
Subgroup analysis of the association between serum uric acid levels and risks of early renal function decline among older people. Odds ratios were calculated by multivariable logistic regression after adjustment for 20 demographic and clinical variables. Serum uric acid levels of 5.0–5.9 mg/dL served as the reference group. Bars denote 95% confidence intervals.

In subgroups analyses, the association between hyperuricemia and early renal function decline remained consistent across all subgroups of interest. There was no significant interaction between SUA levels and subgroups on the risks of ≥ 30% eGFR decline over 2 years (Fig. 3).

In the sensitivity analyses, where excluding participants taking urate-lowering agents or having missing baseline biochemical data, changing the threshold for eGFR to 40%, or re-grouping the participants into deciles, the associations between hyperuricemia and the risks of early renal function decline were consistently significant (Supplemental Tables S1–S3). In the time-to-event analyses, multivariable Cox proportional hazards regression analyses further confirmed that hyperuricemia was associated with 1.12-fold (95% CI = 1.03–1.22, for SUA 6.0–6.9 mg/dL) to 1.6-fold (95% CI = 1.37–1.86, SUA ≥ 10.0 mg/dL) higher hazards to develop early renal function decline. Competing-risk analyses also produced consistent results (Table 3).

**Table 3:** Cox regression and competing-risk analyses for the risks of eGFR decline ≥0% over 2 years in older people.

Serum uric acid (mg/dL)	Cox Regression Analysis		Competing Risk Analysis	
	Adjusted Hazard Ratio (95% CI)^a^	P	Adjusted Hazard Ratio (95% CI)^a^	P
2.0–2.9	1.12 (0.86–1.47)	0.407	1.13 (0.87–1.47)	0.374
3.0–3.9	1.01 (0.87–1.17)	0.898	1.00 (0.87–1.16)	0.955
4.0–4.9	1.01 (0.92–1.11)	0.878	1.00 (0.91–1.10)	0.965
5.0–5.9	Reference		Reference	
6.0–6.9	1.12 (1.03–1.22)	0.007	1.12 (1.03–1.22)	0.006
7.0–7.9	1.19 (1.08–1.30)	<0.001	1.19 (1.08–1.31)	<0.001
8.0–8.9	1.29 (1.16–1.43)	<0.001	1.26 (1.14–1.41)	<0.001
9.0–9.9	1.36 (1.18–1.57)	<0.001	1.31 (1.13–1.51)	<0.001
≥10	1.60 (1.37–1.86)	<0.001	1.47 (1.26–1.73)	<0.001

^a^Adjusted for age, sex, body mass index, smoking, alcohol drinking, comorbidities and all biochemical data in Table 1.

Abbreviation: CI, confidence interval; eGFR, estimated glomerular filtration rate.

SI conversion factors: To convert uric acid value to μmol/L, multiply by 59.485.

The independent association between hyperuricemia and early renal function decline remained consistent either assessing the ≥ 30% eGFR reduction over 1, 3, or 5 years of follow-up periods. However, hypouricemia with SUA levels <3.0 mg/dL was no longer significantly associated with the higher risk of ≥30% eGFR decline over 3 years (aOR = 1.22, 95% CI = 0.72–2.06) and 5 years (aOR = 1.37, 95% CI = 0.75–2.53) as compared to the reference group (Supplemental Tables S4–S6).

## Discussion

To the best our knowledge, the present study is the largest study to date to examine the association between hyperuricemia and early renal function decline among older people. By utilizing a large population-based elderly cohort including 44,078 participants, our study found that hyperuricemia with SUA levels ≥6.0 mg/dL carried a significantly higher risk for early renal function decline in terms of eGFR reduction ≥30% over 2 years. Notably, the risk of hyperuricemia for early renal function decline elevated in parallel with increasing SUA levels. This independent association between hyperuricemia and early renal function decline was consistent across subgroups and remained robust in the sensitivity analyses. Previous studies regarding the prognostic role of SUA on renal function decline yielded conflicting results, which were partly attributable for differences in sample size and the degree of adjustment for possible confounding factors^18–21^. Although most reports controlled age, sex, body weight, blood pressure, and baseline serum creatinine level, some investigators did not adjust for smoking^21^, alcohol consumption^18–21^, history of cardiovascular diseases^18,19^, and fasting glucose levels^19–21^. After fully adjustment for these confounding factors, our study confirmed that hyperuricemia independently predicted early renal function decline with a ≥30% eGFR decline over 2 years. In contrast to our findings, Nacak *et al*. did not find a significant association between renal function decline and the increase of SUA in stage III to IV CKD patients^21^. Our study indicated that hyperuricemia remained an independent predictor for early kidney function decline when SUA ≥7.0 mg/dL for those with baseline eGFR ≥45 mL/min/1.73 m^2^ and when SUA ≥10.0 mg/dL for those with baseline eGFR <45 mL/min/1.73 m^2^ even after extensive adjustment for possible confounding factors. This discrepancy may be explained by the relatively small patient number and absent adjustment of several confounders with kidney disease progression in Nacak’s study^21^. Furthermore, some other previous studies examined the association between quintiles of hyperuricemia and renal function decline^18,19^. This quantile classification approach is of less clinical applicability and also limited to unequal reference SUA levels^18,19^. By contrast, our large-scale cohort study can determine the threshold of SUA level ≥6.0 mg/dL above which the risk for eGFR decline is increased.

The present study also showed that hyperuricemia-associated higher risks for early renal function decline were consistently significant across different strata of baseline kidney function. SUA levels ≥7.0 mg/dL in older people with eGFR 45–60, 60–90 and >90 mL/min/1.73 m^2^ were associated with higher chances for early renal function decline even after adjusting other risks factors for renal disease progression. In those with eGFR <45 mL/min/1.73 m^2^, the risk of early renal function decline did not significantly increase until SUA levels exceeded 10.0 mg/dL. The higher SUA threshold for early renal function decline in the strata of eGFR <45 mL/min/1.73 m^2^ may be explained by that advanced renal dysfunction often accompanies with an increasing prevalence of non-traditional cardiovascular risk factors such as chronic inflammation, oxidative stress and calcium-phosphate imbalance^30^. These non-traditional factors could also result in renal deterioration and attenuate the contribution of hyperuricemia. In accordance to our findings, Liu *et al*. recently discovered that increased SUA had a causal effect in the Chinese older people with eGFR ≥80 mL/min/1.73 m^2^ by a Mendelian randomization approach^31^. Conversely, Hughes *et al*. found that increased SUA due to genetic variants of urate transporters was associated with improved renal function by utilizing a similar genetic statistical analysis^32^. Although Mendelian randomization is a valuable tool by using genetic variants as instrumental variables to test the causal relationship between SUA and eGFR change, this approach presumes that the genetic instruments should have strongly association with SUA levels, affect outcome solely through SUA, and have no association with known confounders^33^. The validity of genetic instruments was only partly verified in Hughes’ study and potentially influenced the association between SUA and renal function^32^. It should be also noted that the cohort analyzed by Hughes *et al*.^32^ were younger than ours. Clearly, further large-scale studies are still required to disentangle the causal mechanism between hyperuricemia and renal dysfunction. Taken together, our study provided important clinical insights that SUA levels ≥7.0 mg/dL in older people with eGFR ≥45 mL/min/1.73 m^2^ and SUA levels ≥10.0 mg/dL in those with eGFR <45 mL/min/1.73 m^2^ should prompt immediate surveillance of kidney function and management of eGFR decline.

Interestingly, in addition to hyperuricemia, low SUA levels <3.0 mg/dL were also associated with higher risks for early renal function decline in older people, suggesting a U-shaped association between SUA levels and eGFR decline. Increasing evidence has suggested that high eGFR is paradoxically associated with increased cardiovascular mortality, probably mediated by malnutrition^34,35^. High eGFR value either indicates a true high GFR or low serum creatinine level. Both SUA and creatinine are markers for nutritional status^36,37^. Malnourished people with both low SUA and creatinine levels can increase infection risks^38^, which probably accounts for higher risks of eGFR decline in our participants of low SUA levels and high eGFR.

The present study had several strengths. First, this is the first large-scale cohort study to examine the association of SUA levels with a novel validated renal endpoint in older people. Utilization of a 44,078-elderly cohort during a 10-year study period provided adequate statistical power. Second, by using a 1-mg/dL SUA increment to classify patients, our results were easily applied for daily practice to risk-stratify older patients. Third, we utilized the CKD-EPI equation, which provides more reliable estimation of renal function^2,30^. Fourth, the independent association between hyperuricemia and high risks for eGFR decline was ascertained in different statistical approaches including multivariable logistic and Cox regression, cubic spline model, sensitivity and subgroup analyses. Nonetheless, several potential limitations should be acknowledged. First, a single baseline SUA level was used to predict early renal progression. Second, albuminuria was determined by the semiquantitative dipstick analysis. However, positive (≥1 + ) and negative dipstick analyses have been reported to well correlate with a urine albumin-to-creatinine ratio ≥30 mg/g or <30 mg/g, respectively^39,40^. Third, the hospitalized or institutionalized older people were not included. Fourth, the medical history of participants was obtained by self-reported questionnaires and information bias cannot be excluded. Nonetheless, agreement between self-reported and hospital-acquired medical record is found to be substantial for diabetes, hypertension, myocardial infarction and stroke^41^. Finally, all study participants were Taiwanese and the conclusions may not be generalized to other ethnicities.

## Conclusion

The present study found that an SUA level ≥6.0 mg/dL was independently associated with early renal function decline with ≥30% reduction in eGFR over 2 years in the elderly. Our data provided the epidemiological evidence that hyperuricemia was an independent risk factor for early decline of kidney function in the older population. Stringent surveillance regarding renal progression should be performed in the older people with hyperuricemia.

## Supplementary information

Supplementary Data

## Data Availability

The data and study materials will not be made available to other researchers for purposes of reproducing the results or replicating the procedure because access to these data is contractually controlled by the Taipei City Hospital and the Department of Health, Taipei City Government. Only analytic methods are available on reasonable request.
