# Supplementary material for: Hyperuricemia Predicts an Early Decline in Renal Function among Older People: A Community-Based Cohort Study
Source: Sci Rep. 2019 Jan 30;9:980. doi: 10.1038/s41598-018-37529-z (PMC6353916; doi:10.1038/s41598-018-37529-z)
Supplement: Supplementary file 1 — Supplementary Data [file 41598_2018_37529_MOESM1_ESM.docx]

**Supplementary Data**

**Hyperuricemia Predicts an Early Decline in Renal Function among Older People: A Community-Based Cohort Study**

**Supplemental Figure S1.** Flow diagram of participant selection.

**Supplemental Table S1.** Sensitivity analyses of the risks of eGFR decline ≥30% over a 2-year follow-up period in older people.

**Supplemental Table S2.** Incidence and risks of eGFR decline ≥40% over a 2-year follow-up period in older people.

**Supplemental Table S3.** Incidence and risks of eGFR decline ≥30% over a 2-year follow-up period in older people stratified by deciles.

**Supplemental Table S4.** Incidence and risks of eGFR decline ≥30% over 1-year follow-up period in older people.

**Supplemental Table S5.** Incidence and risks of eGFR decline ≥30% over 3-year follow-up period in older people.

**Supplemental Table S6.** Incidence and risks of eGFR decline ≥30% over 5-year follow-up period in older people.

**Supplemental Figure S1. Flow diagram of participant selection.**


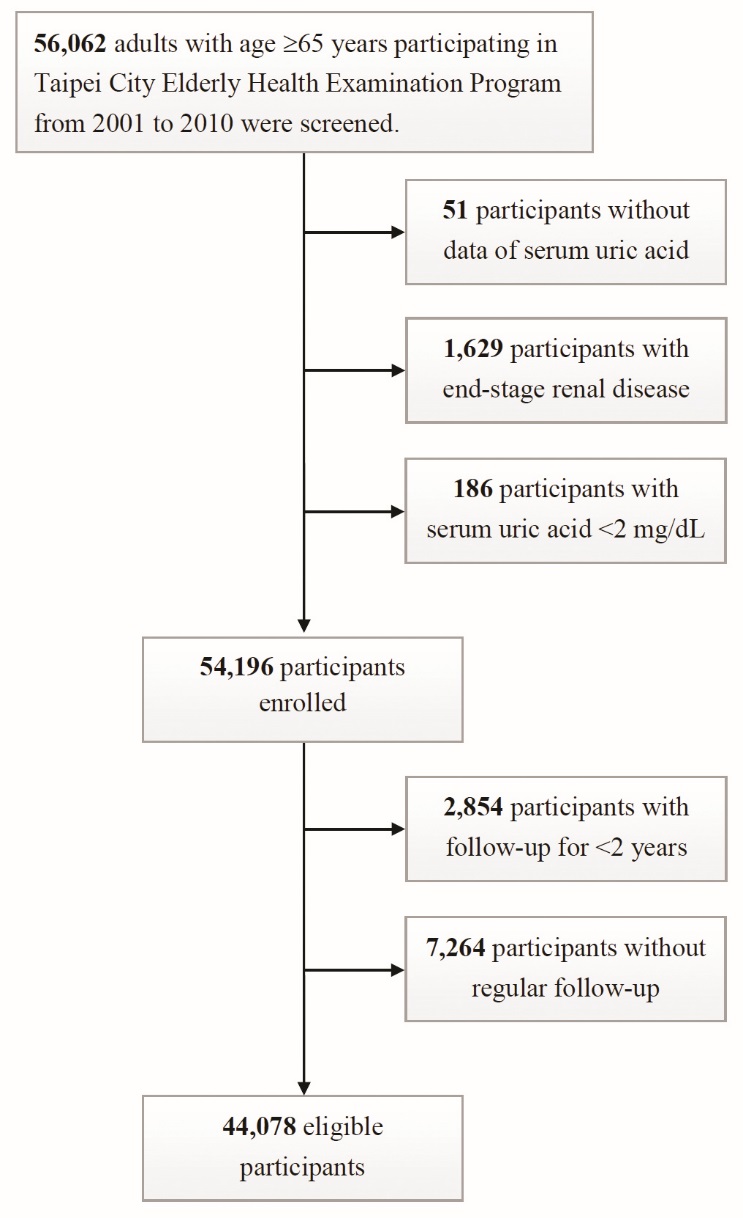


| **Supplemental Table S1. Sensitivity analyses of the risks of eGFR decline ≥30% over a 2-year follow-up period in older people** | | | | | | | | | | | |
| --- | --- | --- | --- | --- | --- | --- | --- | --- | --- | --- | --- |
|  | **Logistic Regression Analysis** | | | | | | | | | | |
|  | **Participants with data of proteinuria** | |  | **Excluding participants with proteinuria or eGFR <60 ml/min** | |  | **Excluding participants taking urate-lowering agents** | |  | **Excluding participants with missing data of covariate** | |
| **Serum uric acid (mg/dL)** | **Adjusted Odds**  **Ratio (95% CI) ^a^** | **P** |  | **Adjusted Odds**  **Ratio (95% CI) ^a^** | **P** |  | **Adjusted Odds**  **Ratio (95% CI) ^a^** | **P** |  | **Adjusted Odds**  **Ratio (95% CI) ^a^** | **P** |
| 2.0-2.9 | 1.76 (0.88-3.51) | 0.108 |  | 1.69 (0.81-3.50) | 0.159 |  | 1.76 (1.13-2.75) | 0.013 |  | 1.68 (1.08-2.62) | 0.022 |
| 3.0-3.9 | 1.02 (0.67-1.56) | 0.929 |  | 0.92 (0.58-1.46) | 0.720 |  | 0.89 (0.67-1.19) | 0.437 |  | 0.90 (0.68-1.20) | 0.475 |
| 4.0-4.9 | 1.11 (0.81-1.53) | 0.510 |  | 1.07 (0.76-1.51) | 0.684 |  | 1.12 (0.93-1.37) | 0.235 |  | 1.10 (0.90-1.33) | 0.351 |
| 5.0-5.9 | Reference |  |  | Reference |  |  | Reference |  |  | Reference |  |
| 6.0-6.9 | 1.35 (0.97-1.87) | 0.075 |  | 1.04 (0.70-1.55) | 0.839 |  | 1.23 (1.02-1.48) | 0.034 |  | 1.21 (1.01-1.46) | 0.044 |
| 7.0-7.9 | 2.29 (1.59-3.28) | <0.001 |  | 2.55 (1.68-3.87) | <0.001 |  | 1.98 (1.63-2.41) | <0.001 |  | 1.92 (1.59-2.33) | <0.001 |
| 8.0-8.9 | 1.95 (1.17-3.26) | 0.010 |  | 2.24 (1.18-4.26) | 0.014 |  | 1.88 (1.47-2.40) | <0.001 |  | 1.78 (1.40-2.28) | <0.001 |
| 9.0-9.9 | 1.62 (0.71-3.71) | 0.255 |  | 1.84 (0.56-6.01) | 0.314 |  | 1.73 (1.22-2.46) | 0.002 |  | 1.62 (1.14-2.31) | 0.007 |
| ≥10 | 7.85 (4.46-13.83) | <0.001 |  | 11.48 (5.02-26.27) | <0.001 |  | 3.38 (2.45-4.68) | <0.001 |  | 3.37 (2.46-4.62) | <0.001 |
| ^a^ Adjusted for age, sex, body mass index, smoking, alcohol drinking, comorbidities and all biochemical data in Table 1.  Abbreviation: CI, confidence interval; eGFR, estimated glomerular filtration rate  SI conversion factors: To convert uric acid value to μmol/L, multiply by 59.485 | | | | | | | | | | | |

| **Supplemental Table S2. Incidence and risks of eGFR decline ≥40% over a 2-year follow-up period in older people** | | | | | | | | | |
| --- | --- | --- | --- | --- | --- | --- | --- | --- | --- |
|  | **Incidence** | |  | | **Logistic Regression Analysis** | | | | |
| **Serum uric acid (mg/dL)** | **No. of Events** | **No. of Participants** | |  | **Crude Odds Ratio (95% CI)** | **P** |  | **Adjusted Odds**  **Ratio (95% CI) ^a^** | **P** |
| 2.0-2.9 | 4 (0.77%) | 522 | |  | 1.39 (0.51-3.85) | 0.521 |  | 1.16 (0.41-3.24) | 0.780 |
| 3.0-3.9 | 14 (0.53%) | 2,656 | |  | 0.96 (0.53-1.71) | 0.882 |  | 0.84 (0.46-1.51) | 0.553 |
| 4.0-4.9 | 47 (0.61%) | 7,708 | |  | 1.11 (0.76-1.62) | 0.599 |  | 1.09 (0.74-1.60) | 0.677 |
| 5.0-5.9 | 61 (0.55%) | 11,076 | |  | Reference |  |  | Reference |  |
| 6.0-6.9 | 61 (0.61%) | 10,047 | |  | 1.10 (0.77-1.57) | 0.589 |  | 1.12 (0.78-1.60) | 0.545 |
| 7.0-7.9 | 69 (1.07%) | 6,435 | |  | 1.96 (1.38-2.77) | <0.001 |  | 1.87 (1.31-2.68) | 0.001 |
| 8.0-8.9 | 33 (0.97%) | 3,387 | |  | 1.78 (1.16-2.72) | 0.008 |  | 1.67 (1.07-2.61) | 0.023 |
| 9.0-9.9 | 9 (0.67%) | 1,344 | |  | 1.22 (0.60-2.46) | 0.583 |  | 0.93 (0.45-1.93) | 0.852 |
| ≥10 | 22 (2.44%) | 903 | |  | 4.51 (2.76-7.38) | <0.001 |  | 2.92 (1.71-4.97) | <0.001 |
| ^a^ Adjusted for age, sex, body mass index, smoking, alcohol drinking, comorbidities and all biochemical data in Table 1.  Abbreviation: CI, confidence interval; eGFR, estimated glomerular filtration rate  SI conversion factors: To convert uric acid value to μmol/L, multiply by 59.485 | | | | | | | | | |

| **Supplemental Table S3. Incidence and risks of eGFR decline ≥30% over a 2-year follow-up period in older people stratified by deciles** | | | | | | | | | |
| --- | --- | --- | --- | --- | --- | --- | --- | --- | --- |
|  | **Incidence** | |  | | **Logistic Regression Analysis** | | | | |
| **Deciles of serum uric acid (range [mg/dL])** | **No. of Events** | **No. of Participants** | |  | **Crude Odds Ratio (95% CI)** | **P** |  | **Adjusted Odds**  **Ratio (95% CI) ^a^** | **P** |
| Decile 1 (2.0-4.2) | 122 (2.47%) | 4,932 | |  | 1.20 (0.91-1.59) | 0.191 |  | 0.89 (0.67-1.18) | 0.426 |
| Decile 2 (4.3-4.7) | 111 (2.76%) | 4,021 | |  | 1.35 (1.01-1.79) | 0.039 |  | 1.10 (0.83-1.47) | 0.500 |
| Decile 3 (4.7-5.2) | 124 (2.42%) | 5,123 | |  | 1.18 (0.89-1.55) | 0.248 |  | 0.99 (0.74-1.31) | 0.956 |
| Decile 4 (5.3-5.6) | 93 (2.09%) | 4,441 | |  | 1.01 (0.76-1.36) | 0.922 |  | 0.92 (0.68-1.24) | 0.574 |
| Decile 5 (5.7-6.0) | 117 (2.57%) | 4,560 | |  | 1.25 (0.94-1.65) | 0.119 |  | 1.18 (0.89-1.57) | 0.244 |
| Decile 6 (6.1-6.4) | 88 (2.06%) | 4,263 | |  | Reference |  |  | Reference |  |
| Decile 7 (6.5-6.8) | 97 (2.57%) | 3,779 | |  | 1.25 (0.93-1.67) | 0.134 |  | 1.31 (0.97-1.75) | 0.077 |
| Decile 8 (6.9-7.4) | 136 (2.96%) | 4,596 | |  | 1.45 (1.10-1.90) | 0.008 |  | 1.56 (1.19-2.06) | 0.001 |
| Decile 9 (7.5-8.2) | 142 (3.53%) | 4,026 | |  | 1.73 (1.32-2.27) | <0.001 |  | 1.92 (1.46-2.53) | <0.001 |
| Decile 10 (8.3-26) | 160 (3.69%) | 4,337 | |  | 1.82 (1.40-2.37) | <0.001 |  | 1.88 (1.43-2.48) | <0.001 |
| ^a^ Adjusted for age, sex, body mass index, smoking, alcohol drinking, comorbidities and all biochemical data in Table 1.  Abbreviation: CI, confidence interval; eGFR, estimated glomerular filtration rate  SI conversion factors: To convert uric acid value to μmol/L, multiply by 59.485 | | | | | | | | | |

| **Supplementary Table S4. Incidence and risks of eGFR decline ≥30% over 1-year follow-up period in older people** | | | | | | | | | |
| --- | --- | --- | --- | --- | --- | --- | --- | --- | --- |
|  | **Incidence** | |  | | **Logistic Regression Analysis** | | | | |
| **Serum uric acid (mg/dL)** | **No. of Events** | **No. of Participants** | |  | **Crude Odds Ratio (95% CI)** | **P** |  | **Adjusted Odds  Ratio (95% CI) ^a^** | **P** |
| 2.0-2.9 | 17 (3.8%) | 444 | |  | 2.16 (1.30-3.58) | 0.003 |  | 1.73 (1.03-2.89) | 0.038 |
| 3.0-3.9 | 43 (1.8%) | 2287 | |  | 1.04 (0.74-1.45) | 0.828 |  | 0.84 (0.60-1.19) | 0.327 |
| 4.0-4.9 | 141 (2.1%) | 6681 | |  | 1.17 (0.93-1.46) | 0.175 |  | 1.04 (0.82-1.30) | 0.766 |
| 5.0-5.9 | 173 (1.8%) | 9546 | |  | Reference |  |  | Reference |  |
| 6.0-6.9 | 160 (1.8%) | 8698 | |  | 1.02 (0.82-1.26) | 0.891 |  | 1.18 (0.95-1.47) | 0.144 |
| 7.0-7.9 | 129 (2.3%) | 5557 | |  | 1.29 (1.02-1.62) | 0.032 |  | 1.64 (1.29-2.08) | <0.001 |
| 8.0-8.9 | 58 (1.9%) | 2936 | |  | 1.09 (0.81-1.47) | 0.566 |  | 1.51 (1.11-2.07) | 0.009 |
| 9.0-9.9 | 27 (2.2%) | 1174 | |  | 1.28 (0.85-1.92) | 0.245 |  | 1.69 (1.11-2.59) | 0.015 |
| ≥10 | 31 (3.9%) | 781 | |  | 2.24 (1.52-3.31) | <0.001 |  | 2.80 (1.87-4.28) | <0.001 |
| ^a^ Adjusted for age, sex, body mass index, smoking, alcohol drinking, comorbidities and all biochemical data in Table 1.  Abbreviation: CI, confidence interval; eGFR, estimated glomerular filtration rate  SI conversion factors: To convert uric acid value to μmol/L, multiply by 59.485 | | | | | | | | | |

| **Supplementary Table S5. Incidence and risks of eGFR decline ≥30% over 3-year follow-up period in older people** | | | | | | | | | |
| --- | --- | --- | --- | --- | --- | --- | --- | --- | --- |
|  | **Incidence** | |  | | **Logistic Regression Analysis** | | | | |
| **Serum uric acid (mg/dL)** | **No. of Events** | **No. of Participants** | |  | **Crude Odds Ratio (95% CI)** | **P** |  | **Adjusted Odds  Ratio (95% CI) ^a^** | **P** |
| 2.0-2.9 | 16 (4.1%) | 390 | |  | 1.42 (0.85-2.38) | 0.184 |  | 1.22 (0.72-2.06) | 0.451 |
| 3.0-3.9 | 52 (2.7%) | 1917 | |  | 0.93 (0.68-1.25) | 0.617 |  | 0.76 (0.55-1.03) | 0.076 |
| 4.0-4.9 | 165 (2.9%) | 5615 | |  | 1.00 (0.82-1.23) | 0.964 |  | 0.91 (0.74-1.12) | 0.361 |
| 5.0-5.9 | 239 (2.9%) | 8170 | |  | Reference |  |  | Reference |  |
| 6.0-6.9 | 202 (2.7%) | 7451 | |  | 0.92 (0.76-1.12) | 0.419 |  | 1.01 (0.83-1.22) | 0.945 |
| 7.0-7.9 | 192 (4.0%) | 4763 | |  | 1.39 (1.15-1.69) | 0.001 |  | 1.56 (1.28-1.91) | <0.001 |
| 8.0-8.9 | 104 (4.0%) | 2537 | |  | 1.42 (1.12-1.79) | 0.004 |  | 1.59 (1.24-2.03) | <0.001 |
| 9.0-9.9 | 35 (3.6%) | 972 | |  | 1.24 (0.86-1.78) | 0.244 |  | 1.29 (0.89-1.89) | 0.181 |
| ≥10 | 59 (9.2%) | 636 | |  | 3.39 (2.52-4.57) | <0.001 |  | 3.00 (2.17-4.15) | <0.001 |
| ^a^ Adjusted for age, sex, body mass index, smoking, alcohol drinking, comorbidities and all biochemical data in Table 1.  Abbreviation: CI, confidence interval; eGFR, estimated glomerular filtration rate  SI conversion factors: To convert uric acid value to μmol/L, multiply by 59.485 | | | | | | | | | |

| **Supplementary Table S6. Incidence and risks of eGFR decline ≥30% over 5-year follow-up period in older people** | | | | | | | | | |
| --- | --- | --- | --- | --- | --- | --- | --- | --- | --- |
|  | **Incidence** | |  | | **Logistic Regression Analysis** | | | | |
| **Serum uric acid (mg/dL)** | **No. of Events** | **No. of Participants** | |  | **Crude Odds Ratio (95% CI)** | **P** |  | **Adjusted Odds  Ratio (95% CI) ^a^** | **P** |
| 2.0-2.9 | 12 (6.4%) | 187 | |  | 1.42 (0.84-2.40) | 0.185 |  | 1.37 (0.75-2.53) | 0.307 |
| 3.0-3.9 | 37 (4.1%) | 897 | |  | 0.97 (0.71-1.33) | 0.871 |  | 0.83 (0.57-1.19) | 0.302 |
| 4.0-4.9 | 123 (4.2%) | 2915 | |  | 0.97 (0.79-1.19) | 0.745 |  | 0.87 (0.69-1.10) | 0.259 |
| 5.0-5.9 | 204 (4.5%) | 4490 | |  | Reference |  |  | Reference |  |
| 6.0-6.9 | 202 (4.7%) | 4265 | |  | 1.06 (0.88-1.27) | 0.539 |  | 1.09 (0.88-1.33) | 0.434 |
| 7.0-7.9 | 168 (6.0%) | 2767 | |  | 1.31 (1.08-1.59) | 0.005 |  | 1.34 (1.08-1.67) | 0.009 |
| 8.0-8.9 | 100 (6.5%) | 1533 | |  | 1.46 (1.17-1.83) | 0.001 |  | 1.47 (1.13-1.90) | 0.004 |
| 9.0-9.9 | 41 (6.9%) | 586 | |  | 1.51 (1.10-2.08) | 0.010 |  | 1.47 (1.02-2.12) | 0.040 |
| ≥10 | 33 (9.3%) | 353 | |  | 2.26 (1.60-3.18) | <0.001 |  | 1.66 (1.10-2.50) | 0.016 |
| ^a^ Adjusted for age, sex, body mass index, smoking, alcohol drinking, comorbidities and all biochemical data in Table 1.  Abbreviation: CI, confidence interval; eGFR, estimated glomerular filtration rate  SI conversion factors: To convert uric acid value to μmol/L, multiply by 59.485 | | | | | | | | | |
